# Fungal consortium of two *Beauveria bassiana* strains increases their virulence, growth, and resistance to stress: A metabolomic approach

**DOI:** 10.1371/journal.pone.0271460

**Published:** 2022-07-14

**Authors:** Andressa Katiski da Costa Stuart, Jason Lee Furuie, Thais Regiani Cataldi, Rodrigo Makowiecky Stuart, Maria Aparecida Cassilha Zawadneak, Carlos Alberto Labate, Ida Chapaval Pimentel

**Affiliations:** 1 Departamento de Patologia Básica, Setor de Ciências Biológicas, Laboratório de Microbiologia e Biologia Molecular (LabMicro), Universidade Federal do Paraná, Curitiba, Paraná, Brazil; 2 Departamento de Genética, Laboratório de Genética de Plantas Max Feffer, Escola Superior de Agronomia Luiz de Queiroz – Esalq/USP, Piracicaba, São Paulo, Brazil; 3 Departamento de Fitotecnia e Fitossanitaríssimo, Programa de Pós-graduação em Agronomia Produção Vegetal, Universidade Federal do Paraná, Curitiba, Paraná, Brazil; Universita degli Studi di Pisa, ITALY

## Abstract

The use of two or more microorganisms in a microbial consortium has been increasingly applied in the biological control of diseases and pests. *Beauveria bassiana* is one of the most widely studied fungal species in biological control, yet little is known about its role in fungal consortiums. In a previous study, our group found that a consortium formed by two strains of *B*. *bassiana* had significantly greater biocontrol potential against the polyphagous caterpillars *Duponchelia fovealis* (Lepidoptera: Crambidae) than either strain on its own. In this study, we use GC-MS and LC-MS/MS to evaluate and discuss the metabolomics of the consortium. A total of 21 consortium biomarkers were identified, corresponding to 14 detected by LC-MS/MS and seven by GC-MS. Antioxidant and anti-inflammatory mechanisms are the main properties of the metabolites produced by the consortium. These metabolites can depress the insect’s immune system, increasing its vulnerability and, hence, the fungal virulence of the consortium. In light of these results, we propose an action model of insect mortality due to the metabolites secreted by the consortium. The model includes the inhibition of defense mechanisms such as pro-inflammatory interleukin secretion, cell migration, cell aggregation, Dif, Dorsal and Relish gene transcription, and JAK/STAT and JNK signaling pathways. It also promotes the cleaning of oxidative molecules, like ROS, NOS, and H_2_O_2_, and the induction of virulence factors.

## Introduction

Entomopathogenic fungi have been successfully used to control agricultural pests. They infect insects by coming into direct contact with their cuticle or when feeding on contaminated leaves or fruits. After contact with the insect, the entomopathogenic fungus penetrates its body cavity, where it grows and colonizes it from within, causing its death [1]. The biological mechanisms involved in the virulence of entomopathogenic fungi include several complex steps ranging from recognition and adhesion to the insect cuticle to the production of a series of molecules. Some of these molecules are hydrolytic and detoxifying enzymes, such as lipases, esterases, proteases, and chitinases. Others include primary and secondary metabolites, including toxins, pigments, and immune suppressors. Fungal infections also result in an increased germination rate and the formation of specialized infection structures like appressoria and blastospores [2–4]. One of the main genera of entomopathogenic fungi is *Beauveria*. Species of this genus produce several toxic metabolites, such as beauvericin and bassianolide, which have the most widely studied insecticidal activity. *Beauveria bassiana* is the most prominent species of the genus, producing other insecticide molecules like tenellin, bassianin, beauverolides, bassiatin, and oosporein [5]. These and other metabolites are very important for the biological control potential of *B*. *bassiana* [6].

Despite recent developments and growing efforts to better understand fungal metabolism and metabolites, much remains unknown [7]. Metabolomics therefore represents an important field for evaluating the metabolites produced or modified by an organism or its relationship with the environment. These metabolites are involved in several chemical reactions within the cell and are part of a complex regulatory system that promotes constant molecular changes. Metabolomics provides integrative information on cell function by detecting the metabolites produced in response to genetic and environmental changes [8–10]. For this and other reasons, it is an excellent alternative for assessing the impact of different variables on certain organisms. The metabolomic approach can be crucial in agriculture as it allows understand the relationship between entomopathogenic microorganisms and their insect hosts. Genomics and transcriptomics have been widely used to elucidate these interactions [11–14]. However, due to the many modifications that happen at the molecular level in these organisms, metabolomics plays an essential role in understanding their actual metabolic panorama at the highest possible level of phenotypically observable phenomena.

Recent studies have shown important advances in biological pest control, with increased pathogenicity when different microorganisms are associated in a microbial consortium [15–19]. Several other benefits have also been reported for plants, soil, and other biomes [20–23] However, despite the enormously important role of microbial consortiums for biocontrol, little is known about their metabolites and microbial interactions. In 2017, Canfora and colleagues [16] evaluated the growth behavior and metabolic response (respiration) of two species of *Beauveria* co-cultivated in different carbon sources. Through the Phenotype MicroArray^®^ technique, they found that sources such as L-Asparagine, L-Aspartic acid, L-Glutamic Acid, m-Erythritol, D-Melezitose, and D-sorbitol cause important changes in the metabolic behavior of fungi when cultivated together, observing a higher metabolic rate than when the fungi were cultivated separately. In addition, SSR markers and qPCR analyses showed that different substrates could promote the growth of one species over another, depending on their ecological niche. This differential growth may be essential for the coexistence of the different species and affect the virulence of the consortium.

Previous studies by our research group have demonstrated that the use of two strains of *B*. *bassiana* (Bov 3 and Bov 2) in a fungal consortium increased the mortality of the pest insect *Duponchelia fovealis* (Lepidoptera: Crambidae) [19]. In the present study, we aim to use untargeted metabolomics with gas and liquid chromatography coupled to mass spectrometers (GC-MS and LC-MS/MS) to evaluate the metabolic alterations caused by the co-cultivation of these strains and to correlate the metabolites produced by this consortium with the increased mortality in *D*. *fovealis* observed by Stuart et al. (2020) [19].

## Material and methods

### Biological material

Two genetically distinct strains of *B*. *bassiana* (Bov 3 and Bov 2) were cultivated in Petri dishes containing Agar Sabouraud culture medium, both separately and co-cultivated to form a fungal consortium [19]. The cultures were incubated in the dark in a biological oxygen demand (BOD) oven for 14 days at 28°C.

### Metabolite extraction

The extraction of fungal metabolites was performed following Hoffman et al. [24] with minor modifications. After the colonies had grown, the mycelium of each treatment (Bov 2, Bov 3, and the fungal consortium) was scraped from the culture medium with a spatula and then macerated separately in liquid nitrogen (N_2_). Extraction was performed in microtubes, from 200 mg of fungal macerate to which 1 mL of 6:2:2 methanol:chloroform:water ice-cold extraction solution was added. These extraction microtubes were vigorously vortexed and placed in an ultrasonic low-temperature bath at 20 Hz s^-1^ for 15 min. The samples were then centrifuged (Eppendorf, Germany) at 4°C for 10 min at 14,000 rpm. Then, the supernatant was filtered using a 0.22 μm Whatman^®^ filter (Merck, Germany) and transferred to a chromatographic vial where the extracts were lyophilized (Thermo Fischer Scientific, MA, USA) until completely dry. Finally, the lyophilized samples were resuspended in 200 μL of extraction solution and aliquoted for use in the GC-MS and LC-MS/MS.

### Identification of metabolites with GC-MS

Each extract aliquot received 10 μL of 1 mg.mL^-1^ solution of the isotopically labeled compounds succinic acid (D4, 98%—DLM 584–5), myristic acid (1, 2, 3–13C3, 99%—CLM 3665–0.5), and palmitic acid (1, 2, 3, 4 – 13C4), which were used as external standards. The samples were then lyophilized once more for subsequent derivatization using 30 μL of a 15 mg·mL^-1^ solution of methoxyamine and pyridine for 16 h at room temperature. The silylation of the samples was performed immediately at room temperature for 1 h by adding 30 μL of MSTFA (N-methyl-trimethylsilyl-trifluoroacetamide) with 1% TMCS (trimethylchlorosilane). Lastly, 30 μL of heptane containing 15 ng.g^-1^ of methylesterase was added.

Data from GC-MS was processed using ChromaTOF 4.32 software to conduct baseline correction, deconvolution, retention index (RI), retention time correction (RT), identification, and alignment of peaks. NIST library version 11 was used for the identification of metabolites. Only metabolites with a score of 700 or above were considered. The intensity of each metabolite was normalized by the total ion count (TIC) of each sample.

The samples were analyzed using GC-MS following Budzinski et al. [25] with minor modifications. A series of n-alkanes (C_12_ –C_40_) were used at this stage to calculate the sample retention index [26]. One microliter of each of the derivatized samples was automatically injected in splitless mode by a CTC Combi Pal Xt Duo autosampler (CTC Analytics AG, Switzerland) into an Agilent 7890A gas chromatographer. The chromatographer was equipped with two fused- silica capillary columns; one column with a 20 m x 0.18 mm chemically bonded with 0.18 μm DB-5 film (Agilent) stationary phase, and the other column with 0.9 m x 0.10 mm chemically bonded with 0.10 μm RX-T 17 film (Restek) stationary phase. The injection temperature was 280°C, with a purge flow of 20 mL·min^-1^. The gas flow through the column was 1 mL·min^-1^, and the column temperature was maintained at 70°C for 2 min, which was increased by 15°C·min^-1^ until it reached 320°C and then held at this temperature for 4 min. The column effluent was introduced into the GC x GC/TOF-MS Pegasus 4D ion source (Leco Corporation, St. Joseph, MI, USA) at a temperature of 250°C. Ions were generated by a 70 eV electron beam at an ionization current of 20 mA and 20 spectra·s^-1^, recorded in the range of 45–800 *m/z*. The detector voltage was 1500 V.

Statistical analyses were performed using the MetaboAnalyst 4.0 online software [27] (available at http://www.metaboanalyst.ca/MetaboAnalyst/). A log^2^ transformation and Pareto scaling were applied to the data. The latter uses the square root of the standard deviation to scale each founded variable [28]. Analysis of variance (ANOVA) and principal component analysis (PCA) were used to find differences in the metabolic profile of each group. Metabolite set enrichment analysis (MSEA) was performed to investigate and assign biologically meaningful patterns to a certain group of significantly enriched metabolites.

### Identification of metabolites with LC-MS/MS

The samples were eluted in 200 μL of extraction solution and vigorously shaken immediately before the LC-MS run. Quercetin (C_15_H_10_O_7_) was used as an external standard. The metabolic profiles were analyzed with Acquity high-performance liquid chromatography coupled to a mass spectrometer (UPLC-MS). Samples were initially injected into the chromatograph with a reversed-phase column (Acquity UPCL HSS 1.7 μm, 2.1×100 mm—Waters Corporation, MA, USA) at a temperature of 35°C. The elution solvents were: A) H_2_O with 0.1% formic acid and B) acetonitrile (ACN) with 0.1% formic acid. The solvent gradient changed over the chromatographic run, starting at 95% solvent A and 5% B. The gradient increased linearly to 75% A and 25% B over the next 6 min. The polarity was reversed to 25% A and 75% B for 6 min, and finally 5% A and 95% B for 1 min. The run lasted a total of 14 min. After the separation in liquid chromatography, the samples were automatically injected and analyzed in an Ultima API high-resolution quadrupole-time off light (Q-TOF) mass spectrometer (Waters Corporation, MA, USA) with electrospray ionization (ESI) in positive and negative mode, under the following conditions: 150°C source temperature, 450°C dissolution temperature, 3 kV capillary voltage, and 35 V cone voltage. The nitrogen flow in the cone was 50 L/hr, and desolvation was 550 L/hr. Spectra were acquired in the range of 100–1200 *m/z*. The equipment was previously calibrated with a 0.05 mM sodium formate standard solution diluted in ACN:H_2_O (90:10), which was also used as a lock-mass, correcting the *m/z* values during the run. The solvent flow was 0.5 mL·min^-1^.

Generated data were pre-processed using MassLynx 4.1 software (Waters Corporation, MA, USA) and then analyzed using MetaboAnalyst 4.0 online software [27]. ANOVA and PCA were performed to assess differences in the metabolic profile of the analyzed groups. The metabolites that distinguished the groups were sent for fragmentation (MS/MS) and identification. Fragmentation was performed under the same conditions as the ionization source described above, using collision energies between 15 and 50 eV. The search for metabolites was performed in the Human Metabolome Database (HMDB) using a mass tolerance of up to 0.1 Da and considering the adduct of [M-H]^-^ for the negative mode and [M+H]^+^ for the positive mode. The structures of the molecules were imported and underwent in silico fragmentation using ACD/MS Structure ID software suite (ACD/labs, Toronto, Canada). The fragmentation profile of each molecule proposed by the program was then compared to the fragments generated by MS/MS to accept or reject the identification of metabolites according to similarity. Molecules with a match of over 90% were considered identified. On the other hand, molecules that did not meet the identification threshold were classified as their corresponding class.

## Results

A total of 397 molecules in the different conditions analyzed (Bov 3, Bov 2, and consortium) were identified by GC-MS and LC-MS/MS. The differences in the metabolic profiles of each molecule are presented below.

### Identification of metabolites with GC-MS

**Fig 1:**
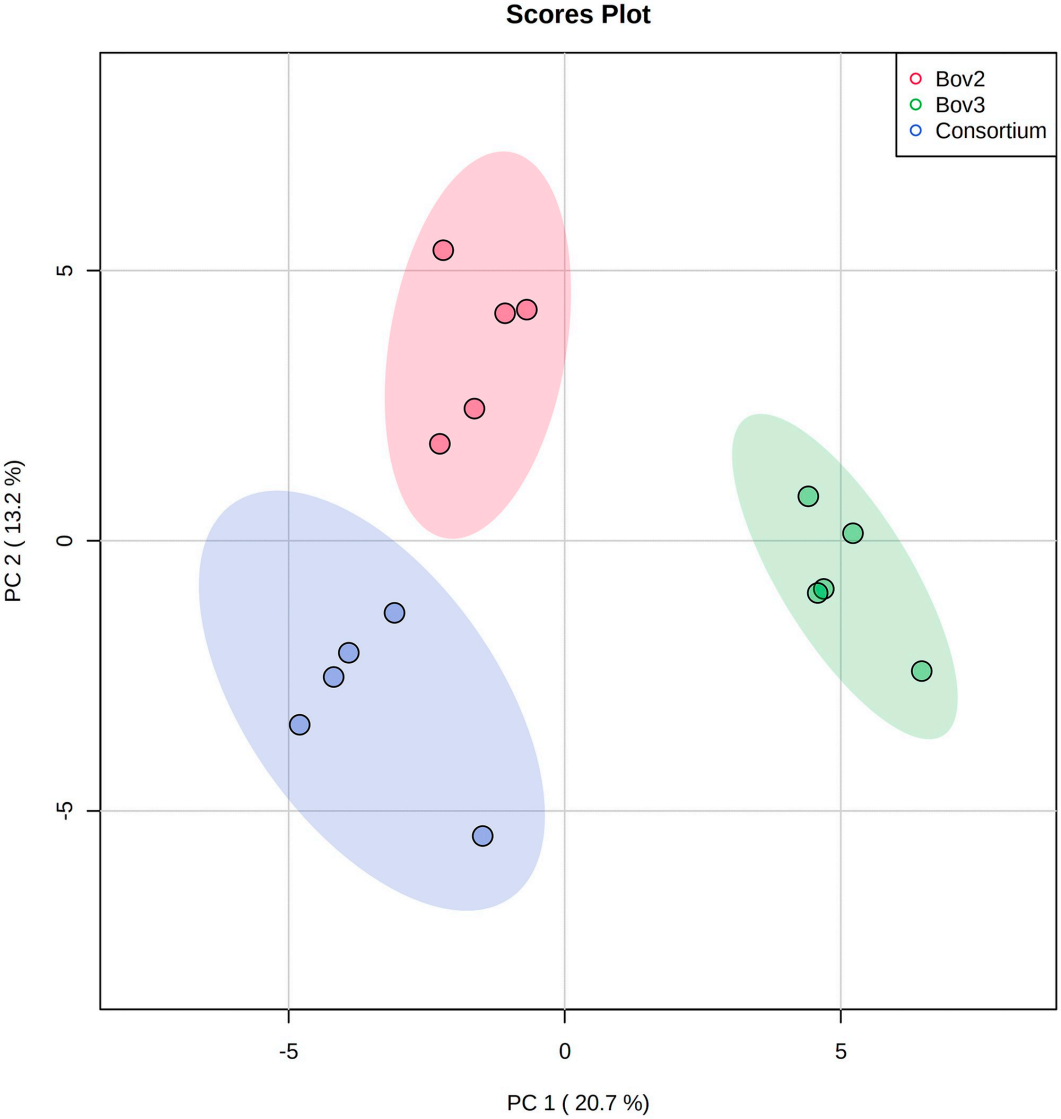
2-D SCORE PLOT of the metabolites identified in Bov 2, Bov 3 and consortium by GC-MS.

**Fig 2:**
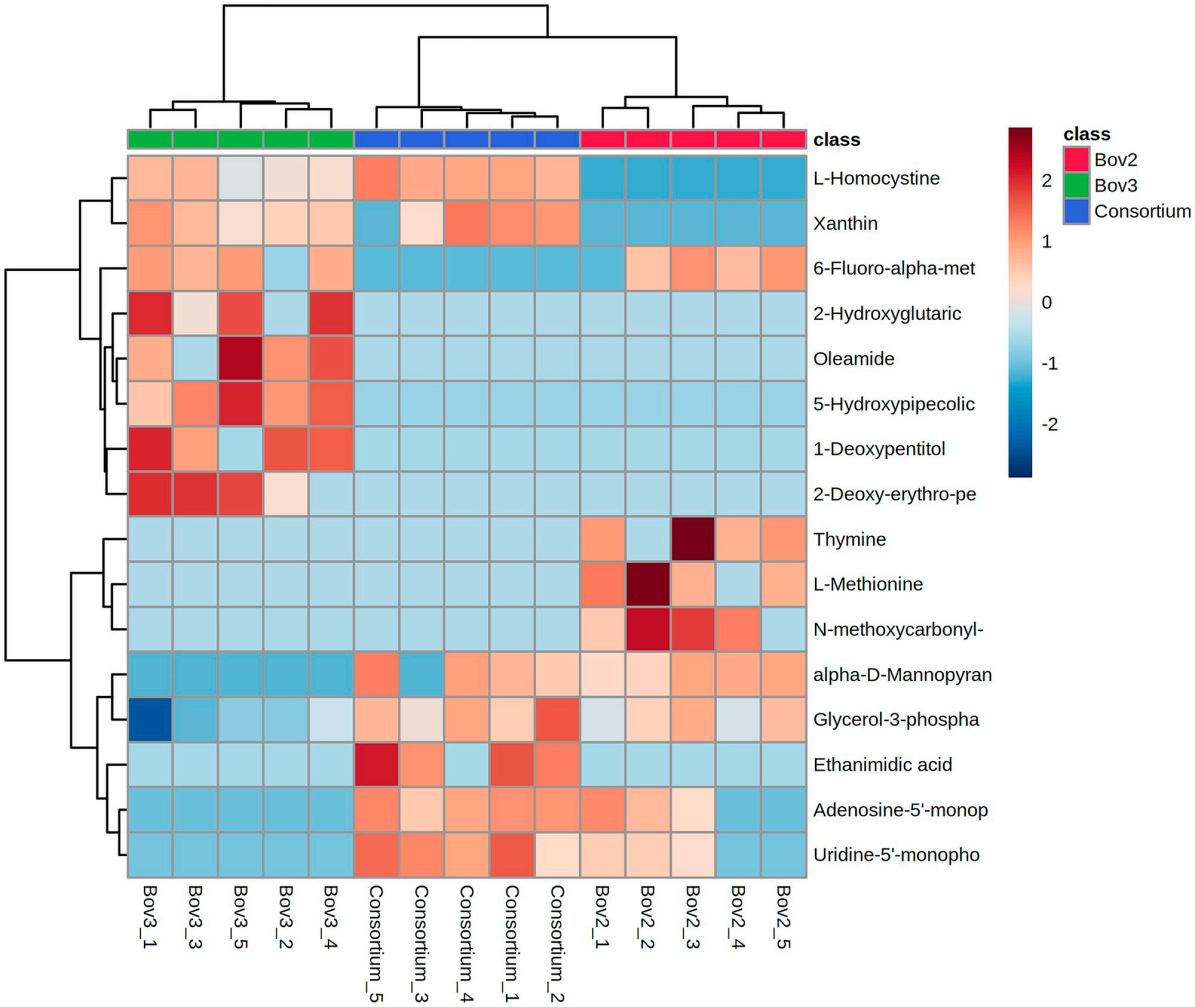
HEATMAP of the 16 biomarkers identified in Bov 2, Bov 3 and consortium.

GC-MS analysis detected a total of 132 molecules. PCA analysis resulted in three well-defined clusters, without outliers, with principal components 1 and 2 (PC1 and PC2) accounting for 33.9% of the variation (Fig 1). The ANOVA identified 16 biomarkers, of which six were significantly (*p≤0*.*001*) more abundant in the consortium, and one was unique to this group (Table 1). The clustering of these 16 biomarkers, demonstrated by the heatmap (Fig 2), indicates closer metabolic profiles between the consortium and Bov 2. However, the metabolic profiles of Bov 2 and the consortium are clearly different, forming distinct groups in the PCA analyses.

**Table 1:** Biomarkers of the consortium.

Metabolite ID	*f*.*value*	*p*.*value*	*-log10(p)*	FDR	Most abundant group
alpha-D-Mannopyranose (D-mannose)	14.655	6.01E-04	3.2213	0.02	Consortium
Adenosine-5’-monophosphate	13.5	8.49E-04	3.0713	0.02	Consortium
L-Homocystine	100.64	3.17E-08	7.4987	0.00	Consortium
Uridine-5’-monophosphate	18.679	2.07E-04	3.6851	0.01	Consortium
Xanthin	11.903	0.001417	2.8486	0.02	Consortium
Glycerol-3-phosphate	11.741	0.0014965	2.8249	0.02	Consortium
Ethanimidic acid	-	-	-	-	Only present in the consortium

f-value: ratio of variances; p-value: significance; FDR: False Discovery Rate

The most abundant metabolites of the consortium are organic compounds of different classes. The D-mannose is classified as a hexose. Adenosine-5’-monophosphate and Uridine-5’-monophosphate are purine and pyrimidine ribonucleoside monophosphates, respectively. The L-Homocystine is known as an L-alpha-amino acid and the xanthin belongs to the class of xanthines. The Glycerol-3’-phosphate is a glycerophosphate (Table 2). There was no known classification for ethanimidic acid in the database consulted (HMDB and PubChem).

**Table 2:** Classification of the metabolites identified in the consortium through GC-MS.

Metabolite	Classification	Description
Alfa-D-Manopiranose (D-manose)	Hexose	Belongs to the class of organic compounds known as hexoses. These are monosaccharides in which the sugar unit is a is a six-carbon containing moeity.
Adenosine-5’-monophosphate	Purine ribonucleoside monophosphates	Adenosine 5’-monophosphate is a purine ribonucleoside 5’-monophosphate having adenine as the nucleobase. Belongs to the class of organic compounds known as purine ribonucleoside monophosphates. These are nucleotides consisting of a purine base linked to a ribose to which one monophosphate group is attached.
L-Homocystine	L-alpha-amino acids	Belongs to the class of organic compounds known as l-alpha-amino acids. These are alpha amino acids which have the L-configuration of the alpha-carbon atom.
Uridine-5’-monophosphate	Pyrimidine ribonucleoside monophosphates	Uridine 5’-monophosphate is a pyrimidine ribonucleoside 5’-monophosphate having uracil as the nucleobase. Belongs to the class of organic compounds known as pyrimidine ribonucleoside monophosphates. These are pyrimidine ribobucleotides with monophosphate group linked to the ribose moiety.
Xanthin	Xanthines	A purine base found in most body tissues and fluids, certain plants, and some urinary calculi. It is an intermediate in the degradation of adenosine monophosphate to uric acid, being formed by oxidation of hypoxanthine. Belongs to the class of organic compounds known as xanthines. These are purine derivatives with a ketone group conjugated at carbons 2 and 6 of the purine moiety.
Glycerol-3-phosphate	Glycerophosphates	Belongs to the class of organic compounds known as glycerophosphates. Glycerophosphates are compounds containing a glycerol linked to a phosphate group.
Ethanimidic acid	-	-

**Fig 3:**
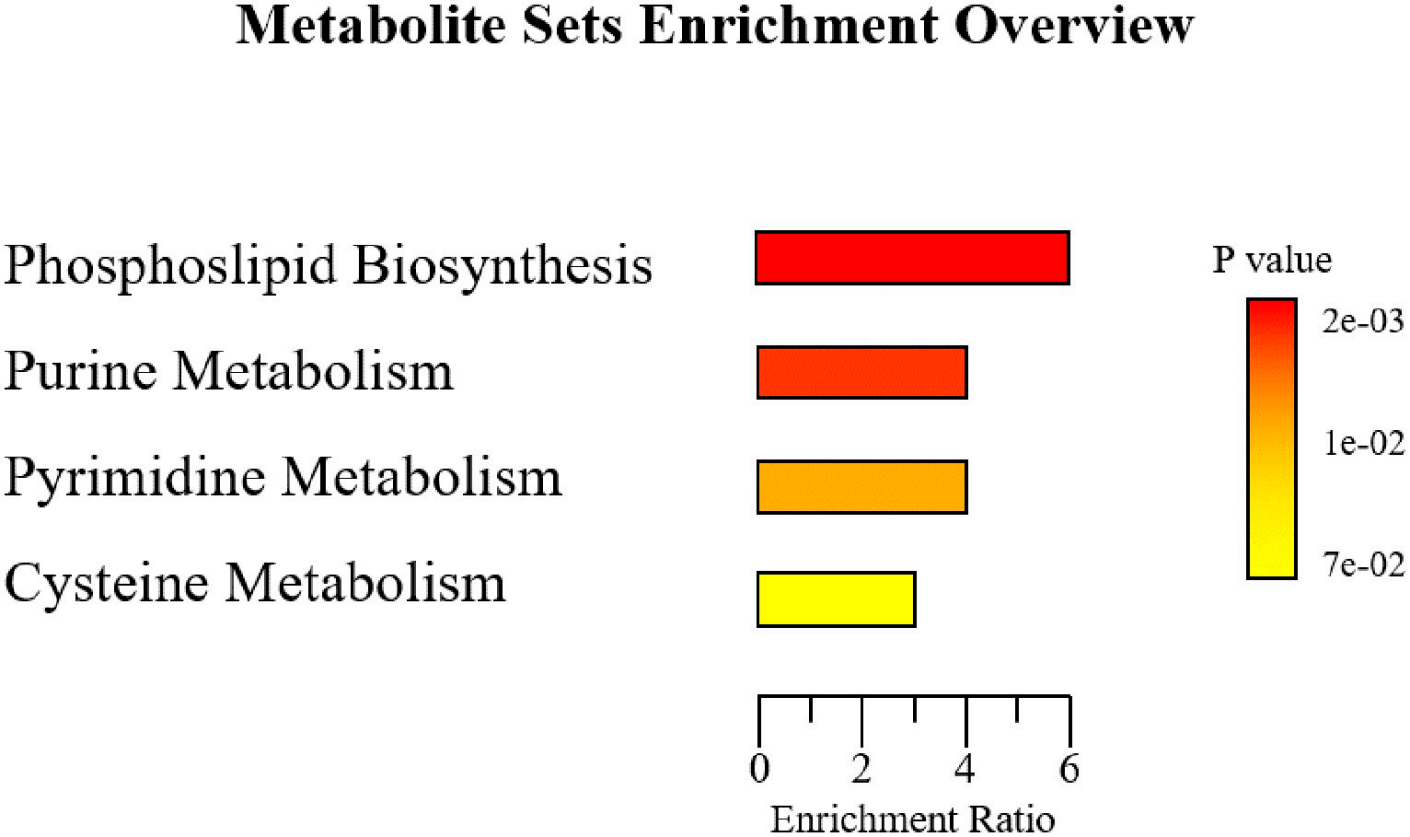
Enrichment ratio of the biosynthetic pathways enriched in the consortium.

The MSEA analysis identified four important pathways that were enriched in the consortium. These are the phospholipid biosynthesis (*p≤0*.*002)*, purine metabolism (*p≤0*.*004*), pyrimidine metabolism *(p≤0*.*02)*, and cysteine metabolism *(p≤0*.*07)* pathways (Table 3). Fig 3 shows the enrichment rate.

**Table 3:** Enriched biosynthetic pathways in the consortium.

Biosynthetic pathway	Hits	HIT molecule	Raw P	Holm P	FDR
Phospholipid biosynthesis	1	Glycerol-3-phosphate	2.73E-03	2.18E-02	2.88E-03
Purine Metabolism	2	Adenosine-5’-monophosphate	4.31E-03	1.25E-01	7.79E-02
		Xanthin			
Pyrimidine metabolism	1	Uridine-5’-monophosphate	1.69E-02	4.74E-01	7.79E-02
Cysteine Metabolism	1	L-Homocystine	7.79E-02	1.00E+00	7.79E-02

Hits: Number of molecules present in the via; Raw-p: p-value; Holm-p: p-value transformed with Holm method; FDR: False Discovery Rate.

### Identification of metabolites with LC-MS/MS

**Fig 4:**
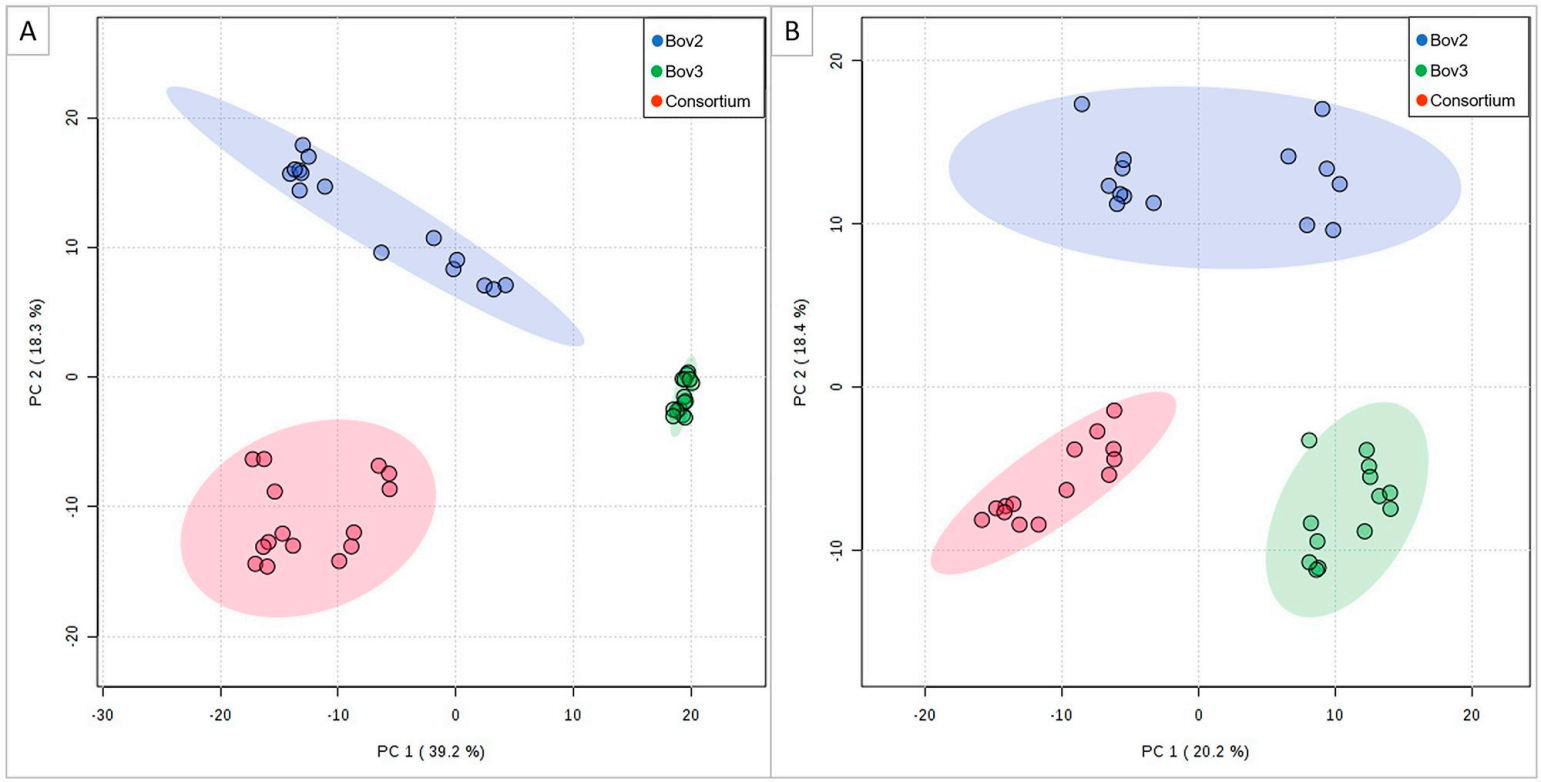
2-D SCORE PLOT of the metabolites identified in Bov 2, Bov 3 and consortium by LC-MS; a) negative ionization mode; b) positive ionization mode.

A total of 138 molecules were obtained in negative mode and 127 in positive mode. The PCA analysis detected separate clusters of metabolites from Bov 2, Bov 3, and the consortium without outliers. Principal components PC1 and PC2 explained 53.1% of the variation in negative mode and 39.2% in positive mode (Fig 4).

Of the biomarkers identified by the ANOVA (123 in negative mode and 83 in positive mode), 48 were selected to perform MS/MS because they were only present in the consortium. The selected metabolites were submitted to fragmentation and *in silico* analysis for identification.

**Fig 5:**
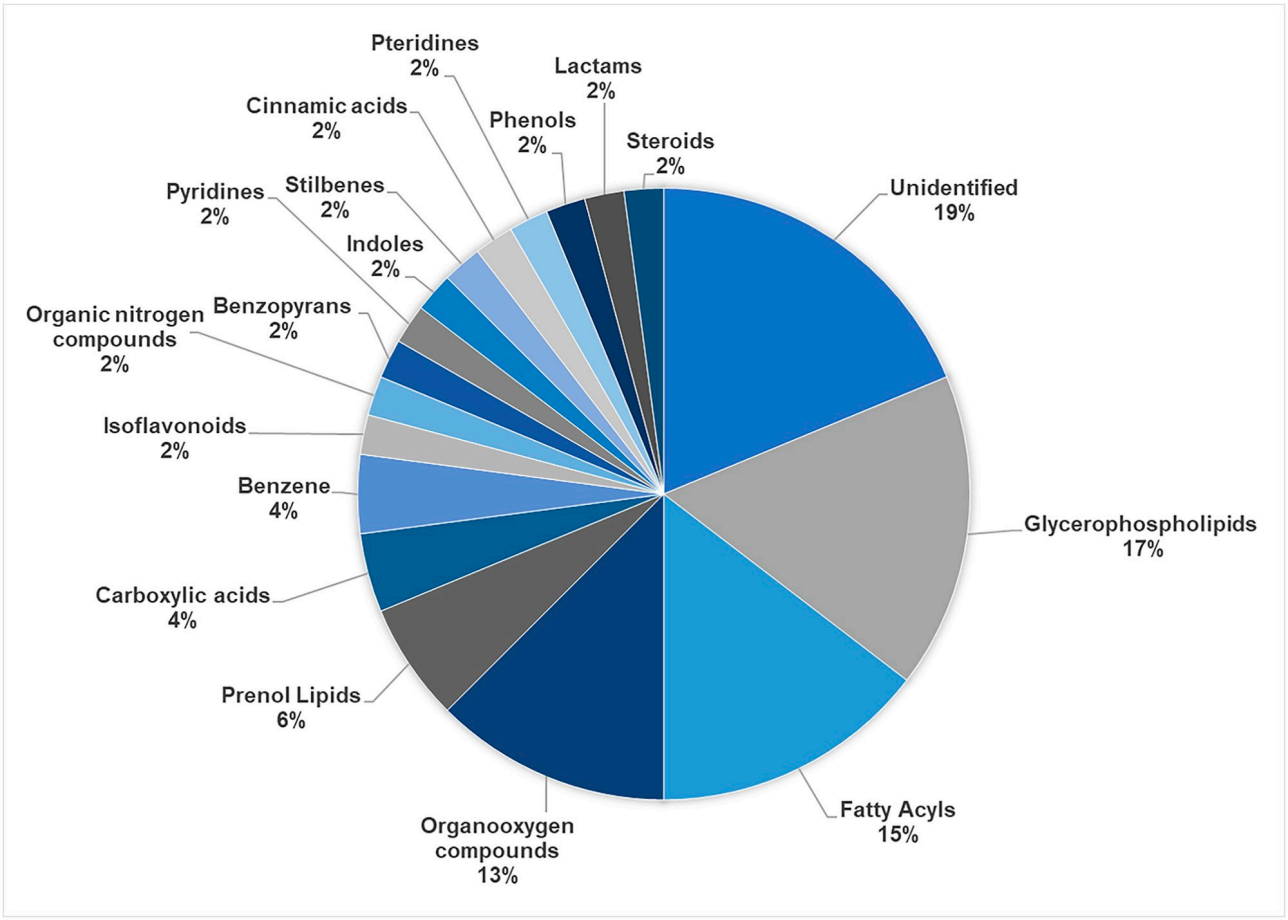
Classes of the metabolites identified in the consortium.

Seventeen classes of metabolites belonging to the consortium were identified (Fig 5). The most representative group was glycerophospholipids (17%), followed by fatty acyls (15%) and organooxygen compounds (13%). Other groups represented between 2% and 6%. Furthermore, 19% of the metabolites could not be identified due to their lack of correlates within the database. Of the 48 metabolites analyzed, 14 met the 90% similarity threshold with the reference compounds in the database (S1 Fig), 39 did not, and nine had no similarity to any metabolite in the database (ND) (Table 4).

**Table 4:** Metabolites identified in the consortium.

RT (min)	*m/z*	Class	HMDB ID	ESI	HMDB CODE	ID (%)
12.10338	433.2322	GPL	LPA(18:2)	(-)	HMDB0007856	99%
11.36876	520.2665	GPL	LPS(18:2)	(-)	HMDB0240604	98%
10.09926	817.3659	GPL	PG(40:8)	(-)	HMDB0010672	86%
12.03293	522.3517	GPL	LPS(18:2)	(+)	HMDB0240604	74%
11.76138	496.3358	GPL	LysoPC(16:0/0:0)	(+)	HMDB0010382	68%
4.402908	500.2827	GPL	LPE(20:5)	(+)	HMDB0011519	64%
8.705952	894.44	GPL	PE(11D5/13M5)	(+)	HMDB0061487	64%
9.2682	632.3954	GPL	PE(28:2)	(+)	HMDB0008855	43%
5.235336	515.1106	OOx	1,4-Di-O-caffeoylquinic acid	(-)	HMDB0034766	100%
7.969968	212.1264	OOx	2-(2,6-dihydroxy-3,4-dimethoxycyclohexylidene)acetonitrile	(-)	HMDB0125517	96%
4.927536	681.291	OOx	Vomifoliol-glu-xyl-glu	(+)	HMDB0038449	71%
3.781152	461.1647	OOx	Verbasoside	(-)	HMDB0039233	66%
5.951484	229.1222	OOx	Talaromycin A	(-)	HMDB0030143	62%
12.7908	829.1768	OOx	Sesaminol 2-O-triglucoside	(-)	HMDB0041775	45%
9.238788	313.2306	FA	8(R)-Hydroperoxylinoleic acid	(+)	HMDB0004706	94%
8.065728	329.2285	FA	9,10,13-TriHOME	(-)	HMDB0004710	93%
11.64373	295.2227	FA	9(10)-EpODE	(+)	HMDB0010220	93%
10.90843	335.2132	FA	Prostaglandin J2	(+)	HMDB0002710	93%
9.651924	313.2345	FA	12,13-DiHOME	(-)	HMDB0004705	89%
10.89612	311.2181	FA	8(R)-Hydroperoxylinoleic acid	(-)	HMDB0004706	85%
9.232632	625.461	FA	FAHFA(22:6/13-O-18:2)	(-)	HMDB0112170	80%
3.543804	525.216	PL	Phaseolus e	(-)	HMDB0035039	100%
12.96385	529.279	PL	Cavipetin A	(-)	HMDB0030363	74%
2.86938	645.3207	PL	Lyciumoside VIII	(-)	HMDB0033210	70%
5.796216	407.1627	BEN	Tamsulosin	(-)	HMDB0014844	97%
10.10473	409.1714	BEN	Tamsulosin	(+)	HMDB0014844	92%
5.732604	466.2853	CBA	N-Arachidonoyl tyrosine	(-)	HMDB0062331	91%
3.33792	586.3051	CBA	2-amino-4-({1-[(carboxymethyl)-C-hydroxycarbonimidoyl]-2-[(2-hydroxy-5-oxo-1,7-diphenylhept-3-en-1-yl)sulfanyl]ethyl}-C-hydroxycarbonimidoyl)butanoic acid	(+)	HMDB0133930	60%
1.488384	232.1442	PYR	Rotundine A	(+)	HMDB0035271	97%
7.792812	331.2396	CA	[10]-Dehydroshogaol	(+)	HMDB0033126	95%
3.420684	466.2249	SB	(E)-Squamosamide	(+)	HMDB0041088	87%
2.922048	425.1385	ONC	Azimsulfuron	(+)	HMDB0033145	84%
8.014428	346.1628	LA	Cephalexin	(-)	HMDB0014707	82%
4.951476	250.143	IND	Melatonin radical	(+)	HMDB0060070	81%
4.260636	573.2097	PT	Tetrahydrofolyl-[Glu](2)	(-)	HMDB0006825	80%
3.281148	308.1483	BP	5-Amino-2,3-dihydro-6-(3-hydroxy-4-methoxy-1-oxobutyl)-2,2-dimethyl-4H-1-benzopyran-4-one	(+)	HMDB0038582	77%
6.394032	439.1608	ISO	Licorisoflavan A	(+)	HMDB0034184	68%
8.513064	857.3761	ST	Olitoriusin	(-)	HMDB0039542	62%
5.57118	1065.396	PHE	TR-Saponin C	(-)	HMDB0035343	34%
4.953528	1031.23	UN	UN	(-)	UN	UN
12.41939	765.1666	UN	UN	(-)	UN	UN
7.073244	187.1297	UN	UN	(-)	UN	UN
5.387184	187.0942	UN	UN	(-)	UN	UN
6.415236	515.1127	UN	UN	(-)	UN	UN
12.39408	295.2232	UN	UN	(-)	UN	UN
3.578688	304.0451	UN	UN	(-)	UN	UN
12.96522	986.5867	UN	UN	(+)	UN	UN
6.393348	401.2116	UN	UN	(+)	UN	UN

GPL: Glycerophospholipids; OOx: Organooxygen compounds; FA: Fatty Acyls; PL: Prenol Lipids; BEN: Benzenes; CBA: Carboxilic acids; PYR: Pyridines; CA: Cinnamic acids; SB: Stilbenes; ONC: Organic Nitrogen Compounds; LA: Lactams; IND: Indoles; PT: Pteridines; BP: Benzopyrans; ISO: Isoflavonoids; ST: Steroids; PHE: Phenols; UN: Unidentified; ESI: *electrospray* ionization mode.

## Discussion

Biological systems are extremely complex, involving thousands of metabolic processes and pathways. Metabolomics is an important tool for providing an integrated view of the functional status of an organism and can show the changes caused by different “analysis conditions” [8–10, 23]. Many organisms involved in the biological control of pests and diseases have been evaluated to better understand and optimize this process [16, 29–32]. Recently, our research group found that by jointly inoculating two different strains of *B*. *bassiana* (Bov 3 and Bov 2) into *D*. *fovealis* caterpillars, insect mortality was considerably higher than when either strain was used separately [19]. Furthermore, by cultivating these fungi together, there was an increase in the production of several important enzymes involved in biological control processes, like chitinase and cellulase. The present study, therefore, aims to evaluate the metabolic alterations caused by a consortium of the Bov3 and Bov2 strains to ascertain the reasons for increased mortality in *D*. *fovealis*.

One of the main defense mechanisms of insects against entomopathogenic fungi like *B*. *bassiana* is the formation of oxidative molecules such as reactive oxygen and nitrogen species (ROS and NOS) [4, 33]. These oxidative molecules can damage important cellular components, including proteins, lipids, carbohydrates, and nucleic acids. In this regard, the production of antioxidant molecules by fungi is extremely important to circumvent the host’s immune system and settle the infection [34, 35].

In this work, after analyzing the metabolomics of the fungal consortium with GC-MS and LC-MS/MS, several molecules with antioxidant action were identified. Xanthine, a carotenoid with high antioxidant activity, is one such example. Carotenoids are pigments widely distributed in nature, and their production by fungi is related to fungal virulence, as are other fungal pigments like melanin [36]. Another molecule found in the consortium, L-homocystine, participates in the cysteine and methionine metabolic pathway (Table 2). This pathway responds to environmental variations, due to its enormous importance in cell physiology; moreover, proteins derived from this metabolic pathway, including thioredoxin and glutathione, play a key role in cellular protection against oxidative stress [34, 37–40]. These molecules are essential for maintaining cellular homeostasis and detoxifying ROS, singlet oxygen molecules, hydrogen peroxide, superoxide anions, and hydroxyl radicals. In addition to its antioxidant action, glutathione affects immune system modulation, cell cycle regulation, vegetative growth, conidia formation, and the virulence of fungi such as *Candida albicans*, *Alternaria brassicicola*, and *B*. *bassiana* [35, 41, 42].

Besides xanthine and L-homocystine, other molecules with antioxidant potential were detected in the consortium. These included ethanimidic acid (EA), which acts by preventing lipid peroxidation and restoring cell homeostasis [43–45]; the highly antioxidant metabolite [10]-dehydroshogaol (10Dhs), which has already been shown to have insecticidal activity against *Spilosoma obliqua* [46]; D-mannose monosaccharide, which demonstrates potent action against hydroxyl radicals, superoxide anions, and 1,1-diphenyl-2-picrylhydrazyl (DPPH) radicals [47, 48] while also representing an excellent carbon source for fungal growth and sporulation [49]. The cell membrane of fungi is rich in mannose, especially during infection, since it confers resistance to the stress caused by the host’s immune system. It is also directly related to the formation of biofilms and cell communication between different pathogens and can serve as a protective barrier in hostile environments [50]. D-mannose is also a precursor of mannitol, one of the most important polyols found in fungi, which acts on carbohydrate storage and translocation, conidia germination under stress situations, osmotic stress resistance, and also as an antioxidant [51, 52]. In *B*. *bassiana*, the deletion of the Sur7 gene, related to stress tolerance, fungal development, and virulence, led to a reduction in mannitol production [53]. Consequently, the authors found that susceptibility to oxidative stress increased and sporulation reduced, demonstrating the molecule’s importance in fungal pathogenesis.

The production of xanthine, L-homocystine, D-mannose, EA, and 10Dhs by the consortium is probably related to the stress situation that occurs due to the co-cultivation of Bov 2 and Bov 3, which induces the production of “defense” metabolites. The antioxidant metabolites reported in this study can thus act expressly by inhibiting oxidative molecules, protecting the pathogen from the insect’s immune system during a potential infection.

Enrichment analysis showed that adenosine 5’-monophosphate (AMP) and xanthine play an important role in activating the purine metabolic pathway (Table 2). In fungi, these molecules have numerous functions, ranging from nucleic acid synthesis (DNA and RNA) to energy metabolism through the synthesis of the main energy storage molecules ATP and GTP. They also play a role in cell signaling by producing cyclic AMP (cAMP) and GMP (cGMP), and cofactors like NADH, NADPH, and coenzyme A [54, 55]. The degradation of purines to obtain nitrogen is a common reaction since it is essential for fungal survival and development. Purine degradation has already been linked to fungal virulence in *C*. *albicans*, which uses purines as a nitrogen source that is essential for adhesion to host tissue and hyphae morphogenesis production [56]. Deletion of genes related to the *de novo* purine biosynthetic pathway has resulted in expressive or complete inhibition of virulence in *C*. *albicans*, *Aspergillus fumigatus*, *Cryptococcus neoformans*, and *Magnaporthe oryzae* [57–59].

Increased levels of AMP induce cAMP production and activates the expression of AMP-activated protein kinase (AMPK). cAMP affects cell signaling, vegetative growth, appressorium formation, cell wall integrity, and fungal pathogenicity. It is also related to the activation of the purine biosynthetic pathway. The deletion of membrane proteins and enzymes that interact with cAMP induced morphological changes and drastically reduced vegetative growth and virulence in the phytopathogenic fungus *M*. *oryzae* [60]. AMPK, in turn, exerts anti-inflammatory activity by suppressing the insect PI3K/Akt and JAK/STAT immune activation pathways [61–65] as well as the anti-inflammatory and antioxidant metabolite 1,4 -Di-O-caffeoylquinic acid (DiOc). DiOc has an important in vitro anti-inflammatory capacity, inhibiting several molecules and cells participating in the inflammatory reaction [66, 67]. A study from Taiwan by Chiou, Chen and Wei [68] showed that caffeoylquinic acids (CAs) inhibit cell migration and reduce proliferative capacity by suppressing the JNK and PI3K/Akt pathways, interfering with the inflammatory response. Previous studies by the same group also showed that CAs can inhibit oxidative stress caused by free radicals at the inflammatory site, thus acting as an antioxidant as well [69]. These data are consistent with Peluso et al. [66], who noticed the anti-inflammatory action of different CAs by in vitro inhibition of cell migration and secretion of superoxide anions (O^2-^). Once O^2-^ secretion and cell migration are inhibited, the inflammatory response and oxidative stress are fatally impaired. The production of CAs and their antioxidant potential have already been described in endophytic fungi isolated from medicinal plants [70].

The innate immune system of insects is extremely similar to the humans, due to the evolutionary conservation of many signaling pathways and key regulatory molecules [71]. In insects, the innate immunity involves several cells and effector molecules, including antimicrobials, signaling, and oxidants (e.g., ROS and NOS, and phenoloxidases–POs), as well as pro-inflammatory molecules like interleukins. There are several similarities among human and insect signaling pathways that activate the innate immune system. In both cases, infection by pathogens leads to activation of toll-like receptors (TLR), triggering intracellular signaling cascades of the inflammatory transcription factors Relish, Dorsal, and Dif, homologous to NF-κβ, and signaling pathways as JAK/STAT and JNK [4, 33, 72]. In insects, hemocytes and crystal cells are responsible for the immune response. Hemocytes are phagocytes capable of recognizing, engulfing, and destroying pathogens. They regulate the expression of antimicrobial molecules (AMMs) and are induced by the expression of homologous NF-κβ genes. Crystal cells, in turn, are responsible for the melanization of hemolymph through the production of POs [33]. Therefore, molecules with anti-inflammatory activity produced by entomopathogenic fungi can suppress pro-inflammatory cells and genes in insects, inhibiting their immune system and increasing fungal virulence.

Other molecules identified in the consortium as having anti-inflammatory potentials are prostaglandin J2 (PGJ2) and the endocannabinoid N-arachidonoyl tyrosine (Table 4).

The production of prostaglandins has been widely described in pathogenic fungi (*A*. *fumigatus*, *Tricophyton rubrum*) and yeasts (*C*. *albicans*, *C*. *neoformans*, and *Hystoplasma capsulatum*). These eicosanoids are directly related to fungal pathogenicity due to their ability to suppress the host’s immune response [73, 74]. Noverr et al. [73] explained that the production of prostaglandins by pathogenic fungi can favor chronic fungal infections by reducing the acute inflammatory response through inhibition of TNF-α, which is an important inflammatory molecule, and induction of IL-10. PGJ2 acts via metabolization to 15-deoxy-D12,14-prostaglandin J2 (15d-PGJ2). 15d-PGJ2 can bind with high affinity to the peroxisome proliferator-activated receptor ɣ (PPARɣ), inhibiting several genes related to the inflammatory cascade such as the NF-κβ gene. This gene suppression leads to an extreme anti-inflammatory reaction [75, 76], which also has cytotoxic, cytostatic, and apoptotic effects [77]. The insect’s ecdysone-75 and 78 (E75 and E78) receptors are homologous to PPARɣ, with functional homology demonstrated in several pharmaceutical studies [78–81]. Furthermore, 15d-PGJ2 can block the inflammatory cascade by acting independently from PPARɣ and inhibiting NF-κβ through the suppression of Iκβ kinase [77]. The action of 15d-PGJ2 on insects was demonstrated by Yun et al. [82]. Thus, we can assume that the metabolized PGJ2 produced by the consortium would be able to bind to both E75 and E78 receptors and act independently, inducing apoptosis and inhibiting the insect inflammatory cascade triggered by the fungal infection. These independent mechanisms facilitate the settlement of the infection and increase fungal pathogenicity.

Endocannabinoids like the N-Arachidonoyl tyrosine are, in turn, endogenous products that can regulate different steps of the inflammatory process, such as cell adhesion, vascular tone, and gene expression. The N-Arachidonoyl DOPA (NADA), to which the N-Arachidonoyl tyrosine is a precursor, has been widely studied for its anti-inflammatory effect. NADA is a TLR 2 and TLR 4 agonist that directly reduces endothelial cell recruitment. The TLR-2 and TLR-4 receptors are activated by binding to microbial molecules, triggering an immunological cascade to produce several cytokines for cell communication and recruitment. When NADA binds to these receptors, the cascade is inhibited, impairing the inflammatory reaction [83]. It also has affinity to the PPARɣ receptor, blocking it and further acting as an anti-inflammatory agent [84].

The antioxidant and anti-inflammatory actions of the molecules produced by the consortium may be directly associated with the higher mortality of *D*. *fovealis* caterpillars previously observed by our group [19] since both activities favor infection and increase fungal virulence.

Our study shows that the pyrimidine metabolic pathway is also enriched by the consortium (Fig 3). As for the purines, the pyrimidines have a crucial role in several cellular processes, including the synthesis of DNA, RNA, polysaccharides, glycoproteins, and phospholipids. In addition, pyrimidines play an important part in membrane lipid synthesis and the production of intermediary molecules used in cellular communication. Their metabolic pathway is represented in the consortium by one of its most important molecules, the uridine-5’-monophosphate (UMP) (Table 2), which is an all-pyrimidine nucleotide precursor [85, 86].

Like the pyrimidine pathway, the phospholipid synthetic pathway is also enriched in the consortium and is represented by the metabolite glycerol-3-phosphate (G3P) (Table 2). This synthetic pathway produces membrane glycerophospholipids, which were the most representative metabolites identified by LC-MS/MS (17%—Fig 5). Metabolites identified as LPA (lysophosphatidic acid), LPS (lysophosphatidylserine), and LysoPC (lysophosphatidylcholine), for instance, are all glycerophospholipids (Table 3). Besides being membrane constituents, LPA, LPS, and LysoPC are active in cell signaling, acting as lipid mediators similar to growth factors, controlling apoptosis and cell differentiation [87], and increasing virulence in fungi [88]. LPA participates in the synthesis of lipid droplets, which are involved in stress responses. Its link to fungal virulence is due in part to the viability of conidia, the formation of infection structures such as appressoria and blastospores, as well as penetration and proliferation within the insect [89, 90]. Glycerophospholipids are also an important source of metabolic energy, acting as an energy reservoir [91, 92].

The second most representative class of molecules type in the consortium was fatty acyls (15%—Fig 5), represented by 8(R)-hydroperoxylinoleic acid (8(R)-HPODE), 9,10,13-TriHOME, 9(10)-EpODE, and prostaglandin J2. Fatty acyl molecules are the main component of complex lipids and therefore represent one of the most important types of biological lipids [88]. They are also known as oxylipins and participate in the linoleic acid metabolic pathway, which is directly related to sporulation and spore germination [93, 94]. Oxylipins are derived from oleic, linolenic, and linoleic acids found mainly in glycerophospholipids and act as regulators of fungal growth, cell differentiation, and apoptosis. They are also involved in the infectious disease processes of pathogenic fungi. Oxylipin production is directly related to their adaptability to environmental changes, often induced by oxidative stress and the presence of free radicals. Furthermore, they can behave in a hormone-like manner, signaling and modulating fungal reproduction through the balance between asexual and sexual sporulation, as well as affecting toxin production [95] and fungal pathogenicity [96]. Oxylipins may thus be related to sexual reproduction within the consortium since they comprise two different strains of *B*. *bassiana*, suggesting heterothallism.

In the consortium, glycerophospholipids and fatty acyls may act as virulence factors since they are directly related to cell viability, appressorium and blastopore formation, sporulation, germination, and toxin production, which are all components of the virulence of entomopathogenic fungi [2–4].

## Conclusion

**Fig 6:**
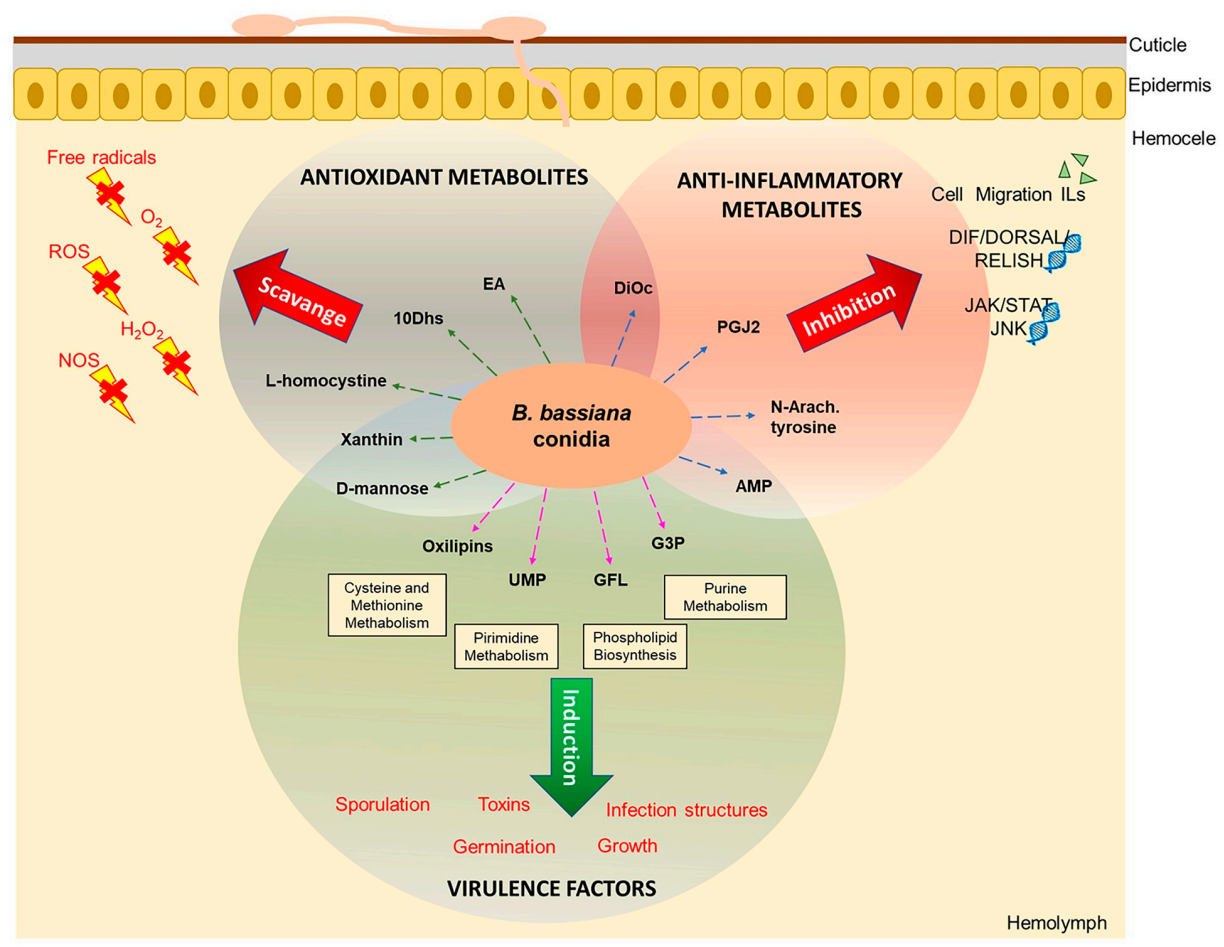
Mode of action hypothesis of the metabolites identified in the consortium on the *Duponchelia fovealis* immune system.

By metabolomics analysis, we identified 21 biomarkers in the consortium. The molecules found have mainly antioxidant and anti-inflammatory effects and may suppress the insect’s immune system. As a result, fungal virulence and pathogenicity increase, leading to greater effectiveness of the consortium in biological control, as observed in previous studies by our group. In addition, diverse mechanisms like toxin production, induction of vegetative growth, induction of germination, sporulation, and formation of infection structures were identified for the metabolites produced by the consortium as increasing its virulence (Fig 6). All the data corroborate the hypothesis that different strains of *B*. *bassiana* growing together in a consortium positively affect the production of molecules that increase lethality to a greater degree than if the strains are used separately. Therefore, this consortium represents an excellent option for the biological control of agricultural pests. Additional studies will be able to assess its action directly on insects.

## Supporting information

S1 Fig ESI-MS/MS spectra of the metabolites identified in the consortium. (PDF)
