# Supplementary material for: Fungal consortium of two Beauveria bassiana strains increases their virulence, growth, and resistance to stress: A metabolomic approach
Source: PLoS One. 2022 Jul 14;17(7):e0271460. doi: 10.1371/journal.pone.0271460 (PMC9282594; doi:10.1371/journal.pone.0271460)

Supplementary figure 1. ESI-MS/MS spectra of the metabolites identified in *B. bassiana* consortium. A) [M-H] of the LPA(18:2) (*m/z* 433) belonging to Glycerophospholipids class; B) [M-H] of the LPS(18:2) *(m/z* 520) belonging to Glycerophospholipids class; Continue.

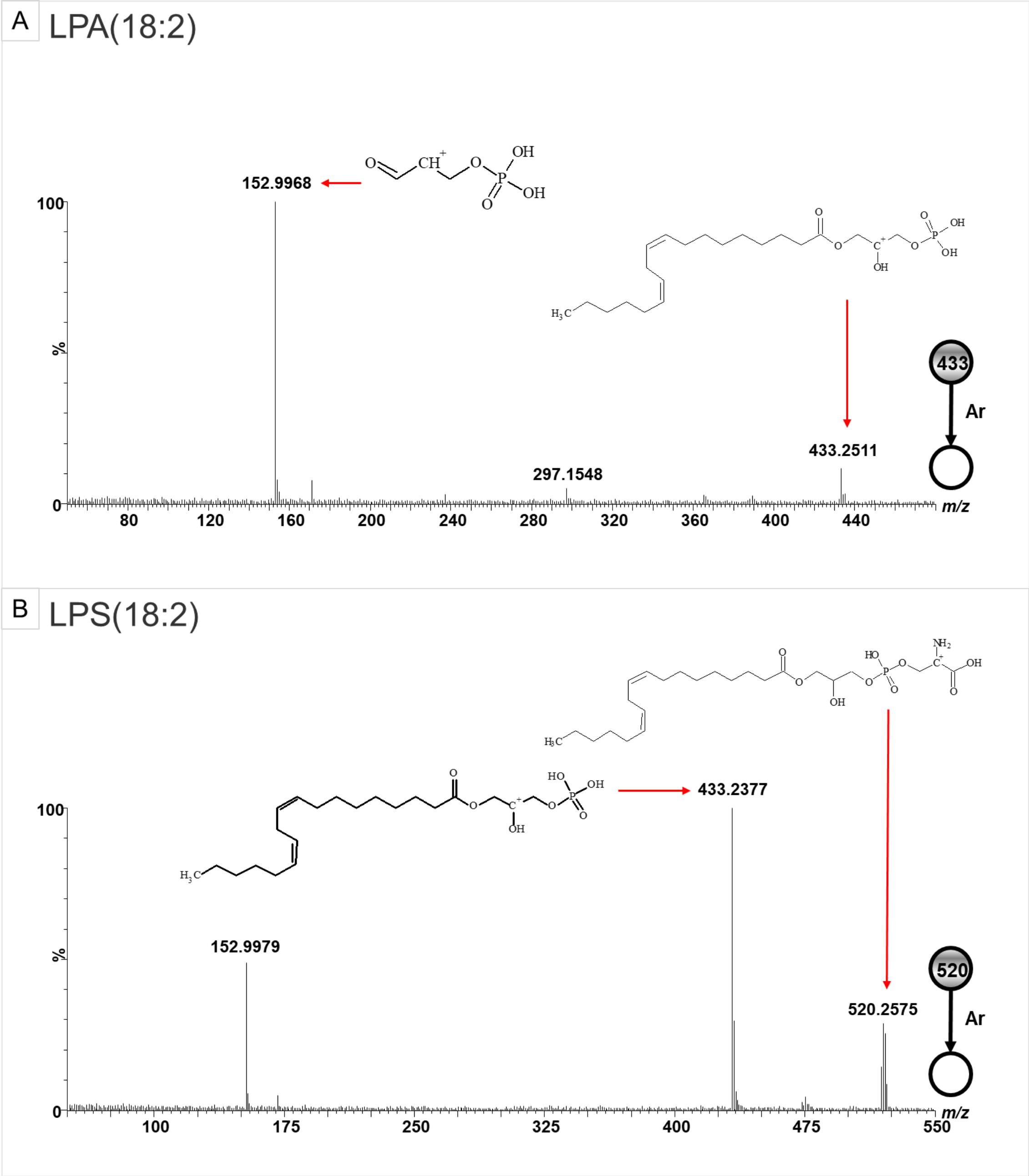

Continuance. Supplementary figure 1. ESI-MS/MS spectra of the metabolites identified in *B. bassiana* consortium. C) [M-H] of the 1,4-Di-O-caffeoylquinic acid *(m/z* 515) belonging to Organooxygen compounds class; D) [M-H] of 2-(2,6-dihydroxy-3,4-dimethoxycyclohexylidene)acetonitrile (*m/z* 212) belonging to Organooxygen compounds class; Continue.

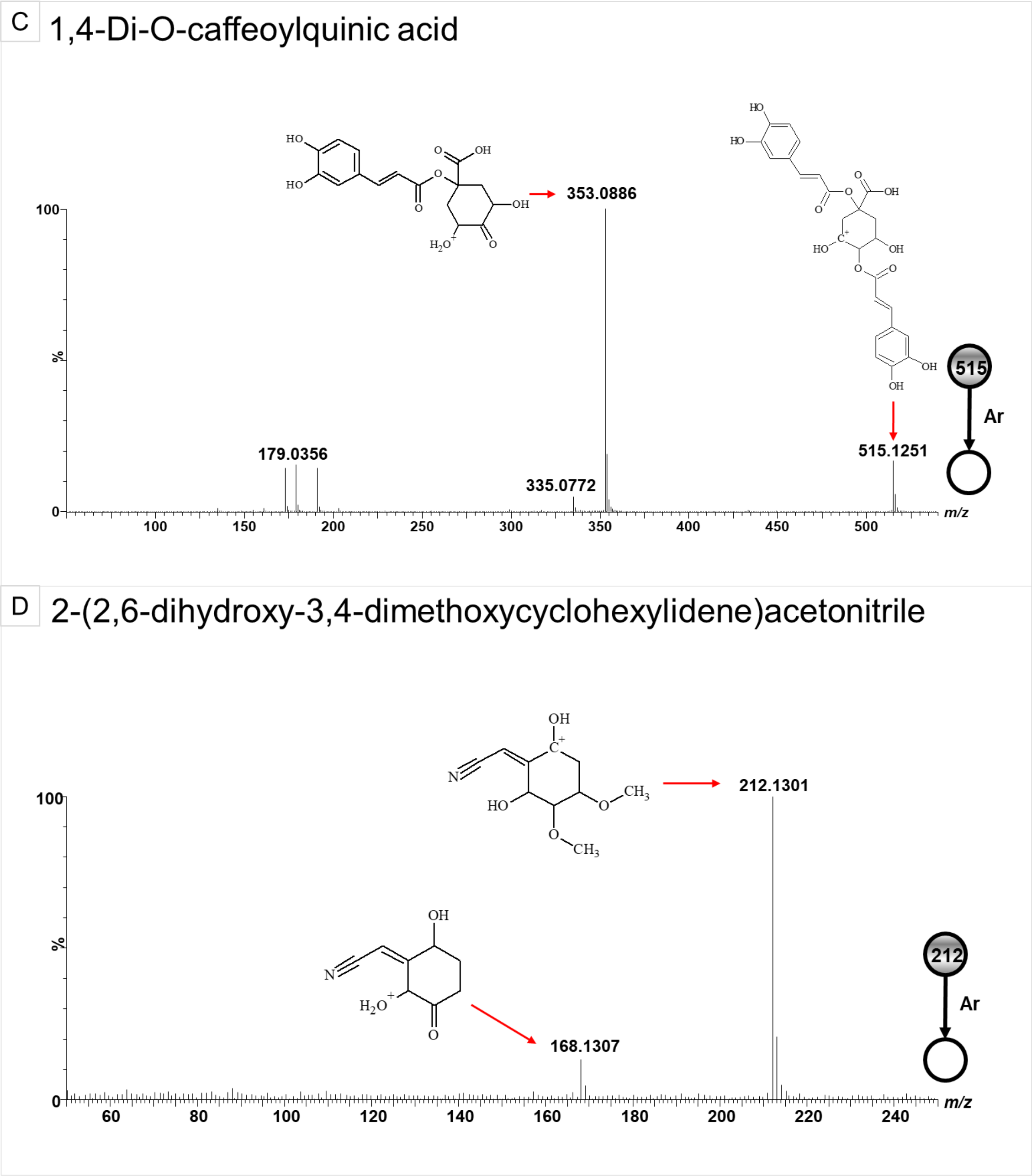

Continuance. Supplementary figure 1. ESI-MS/MS spectra of the metabolites identified in *B. bassiana* consortium. E) [M+H] of the 8(R)-Hydroperoxylinoleic acid (*m/z* 313) belonging to Fatty Acyls class; F) [M-H] of 9,10,13-TriHOME (*m/z* 329) belonging to Fatty Acyls class; Continue

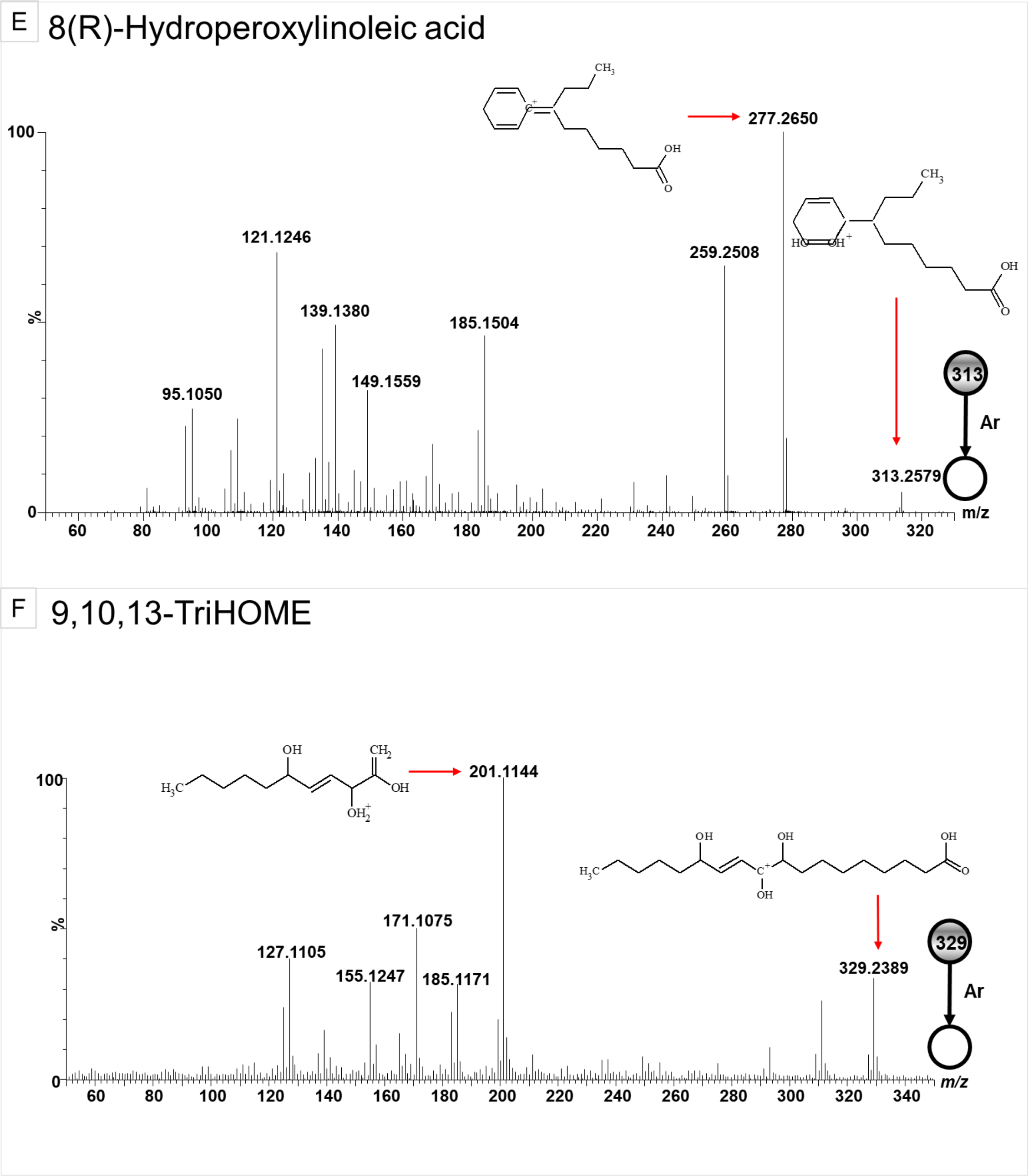

Continuance. Supplementary figure 1. ESI-MS/MS spectra of the metabolites identified in *B. bassiana* consortium. G) [M+H] of the 9(10)-EpODE *(m/z* 295) belonging to Fatty Acyls class; H) [M+H] of the Prostaglandin J2 (*m/z* 335) belonging to Fatty Acyls class; Continue

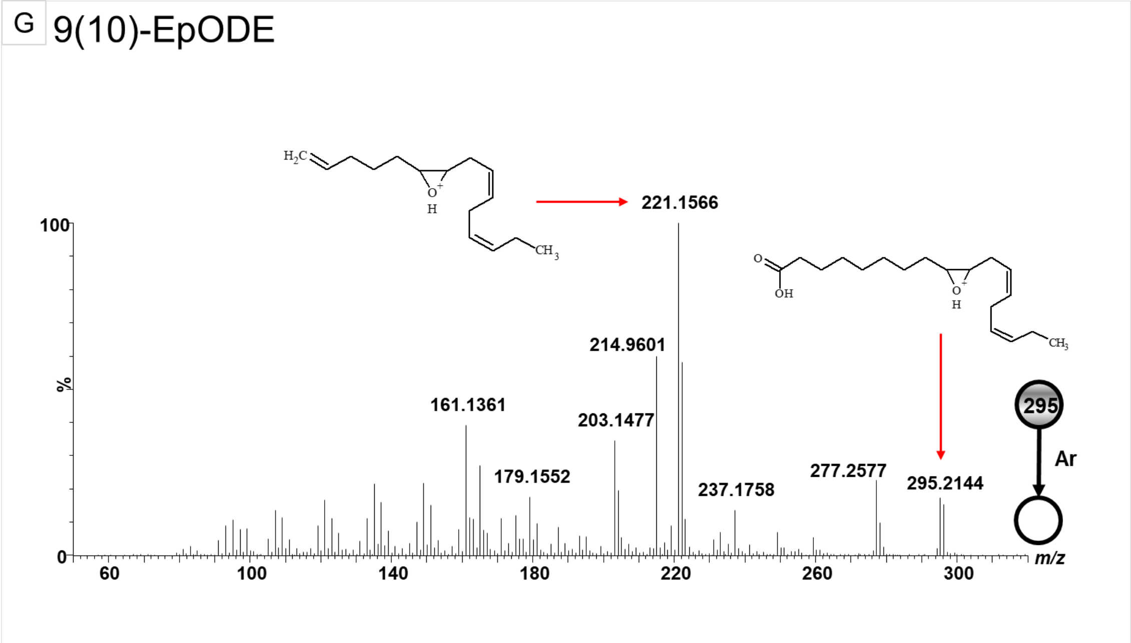

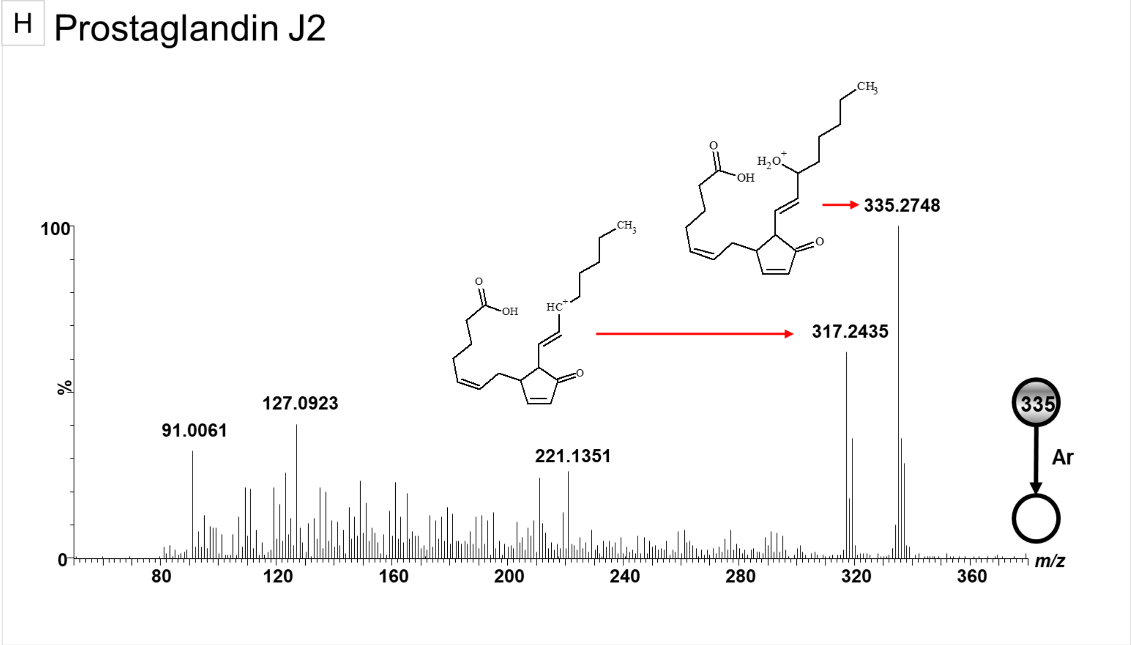

Continuance. Supplementary figure 1. ESI-MS/MS spectra of the metabolites identified in *B. bassiana* consortium. I) [M-H] of the Phaseolus e *(m/z* 525) belonging to Prenol Lipids class; J) [M-H] of the Tamsulosin (*m/z* 407) belonging to Benzenes class; Continue.

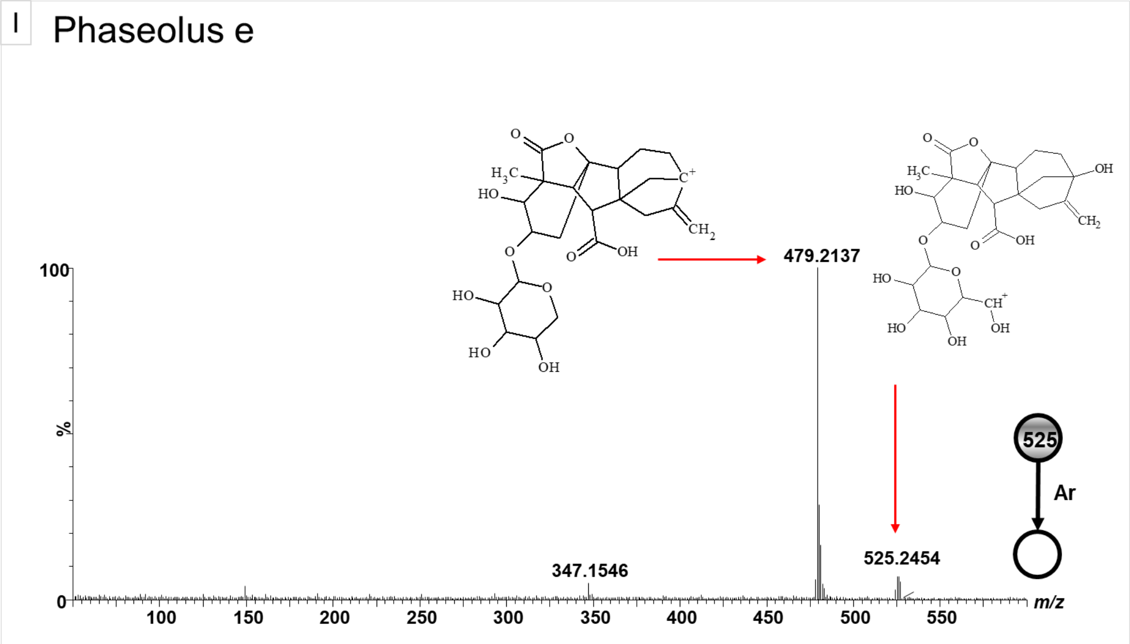

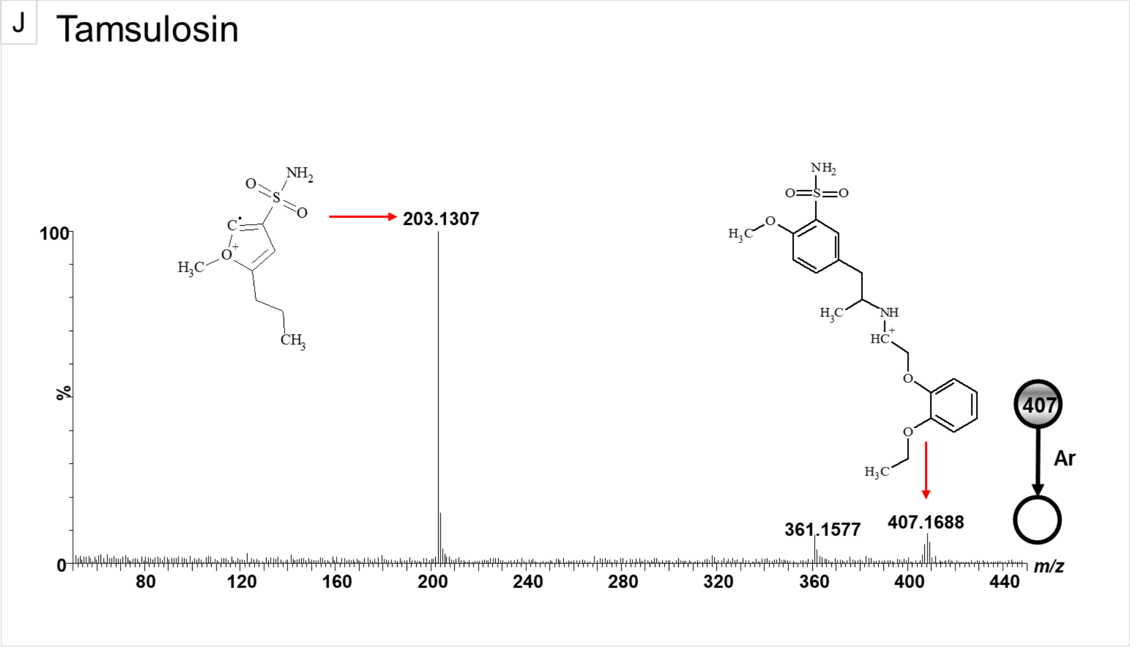

Continuance. Supplementary figure 1. ESI-MS/MS spectra of the metabolites identified in *B. bassiana* consortium. K) [M-H] of the N-Arachidonoyl tyrosine *(m/z* 466) belonging to Carboxylic acids class; L) [M+H] of the Rotundine A (*m/z* 232) belonging to Pyridines class; Continue.

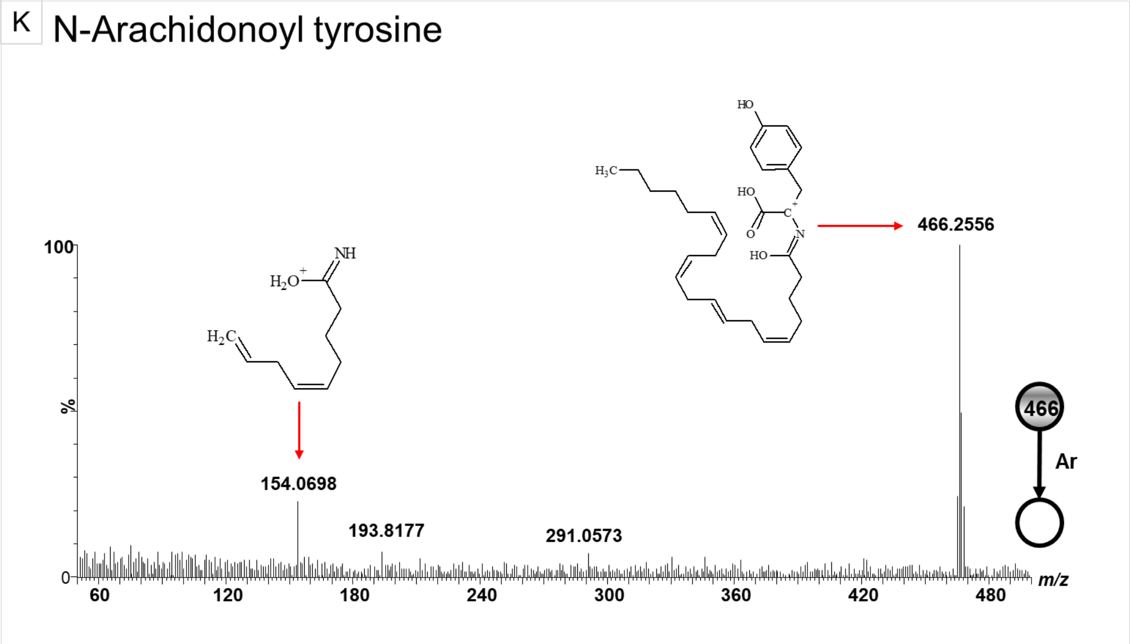

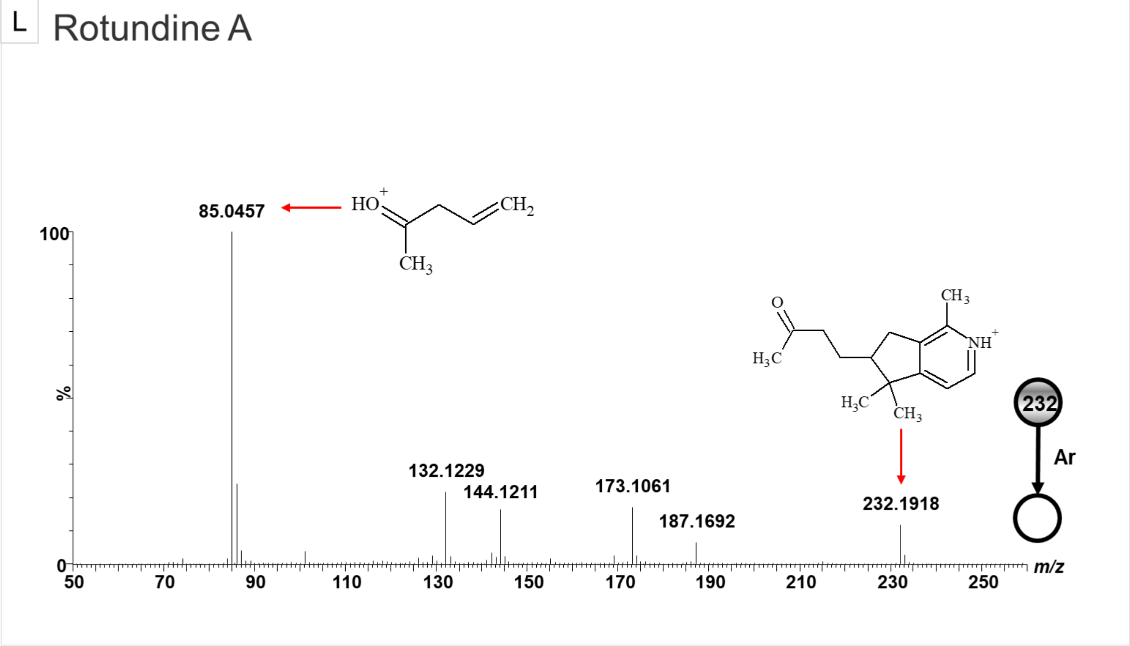

Continuance. Supplementary figure 1. ESI-MS/MS spectra of the metabolites identified in *B. bassiana* consortium. M) [M+H] of [10]-Dehydroshogaol *(m/z* 331) belonging to Cinnamic acids class.

M [10]-Dehydroshogaol

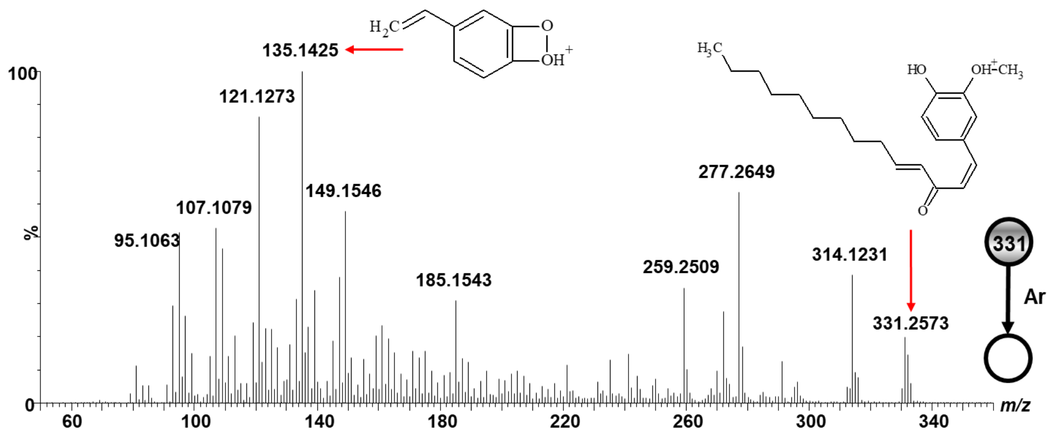

Supplement: S1 Fig — (PDF) [file pone.0271460.s001.pdf]
